# Menstrual hygiene management in flood-affected Bangladesh: addressing socio-cultural barriers, infrastructure gaps, and policy responses

**DOI:** 10.3389/fpubh.2025.1538447

**Published:** 2025-04-04

**Authors:** Md. Al-Mamun, Abul Kalam, Md. Zawadul Karim, Marufa Alam, Tauhid Hossain Khan

**Affiliations:** ^1^BRAC Institute of Governance and Development (BIGD), BRAC University, Dhaka, Bangladesh; ^2^Department of Sociology, Gopalganj Science and Technology University, Gopalganj, Bangladesh; ^3^Rights Jessore, Jashore, Bangladesh; ^4^Department of Sociology, Jagannath University, Dhaka, Bangladesh

**Keywords:** menstrual hygiene management (MHM), floods and menstrual health, women and girls, menstrual hygiene in emergencies, Bangladesh

## Abstract

Women and adolescent girls in flood-prone regions of Bangladesh face significant challenges in managing menstrual hygiene, which severely impacts their health, dignity, and well-being. This study investigates the socio-cultural, infrastructural, and policy barriers to menstrual hygiene management (MHM) during floods in the districts of Noakhali, Feni, Barisal, Khulna, and Satkhira. Conducted from June to October 2024, the research employed a purposive sampling approach, with 30 in-depth interviews (IDIs) conducted with women and adolescent girls directly affected by the floods. Additionally, 12 key informant interviews (KIIs) were carried out with healthcare workers, NGO representatives, and policymakers, alongside field observations in temporary shelters and relief centers. The findings highlight critical barriers, including the lack of accessible and private sanitation facilities in shelters, compounded by inadequate supplies of menstrual hygiene products. In temporary shelters, the absence of specialized toilets and waste disposal systems for menstruation forces women to resort to unhygienic alternatives such as cloth, leaves, or newspapers, leading to increased health risks. Socio-cultural taboos surrounding menstruation further restrict access to proper hygiene materials and support, intensifying the challenges. Moreover, disaster management systems fail to incorporate menstrual hygiene needs, leaving women and girls particularly vulnerable during floods. The study recommends integrating gender-responsive disaster management policies and comprehensive menstrual health education into disaster relief efforts. The Minimum Initial Service Package (MISP) should be utilized to provide urgent reproductive health services. These findings are crucial for policymakers, healthcare professionals, and researchers, and underscore the importance of reducing stigma and promoting dignity for women and girls. By addressing these gaps, Bangladesh can enhance the resilience and health of women and adolescent girls in the aftermath of floods.

## 1 Introduction

**Figure 1:**
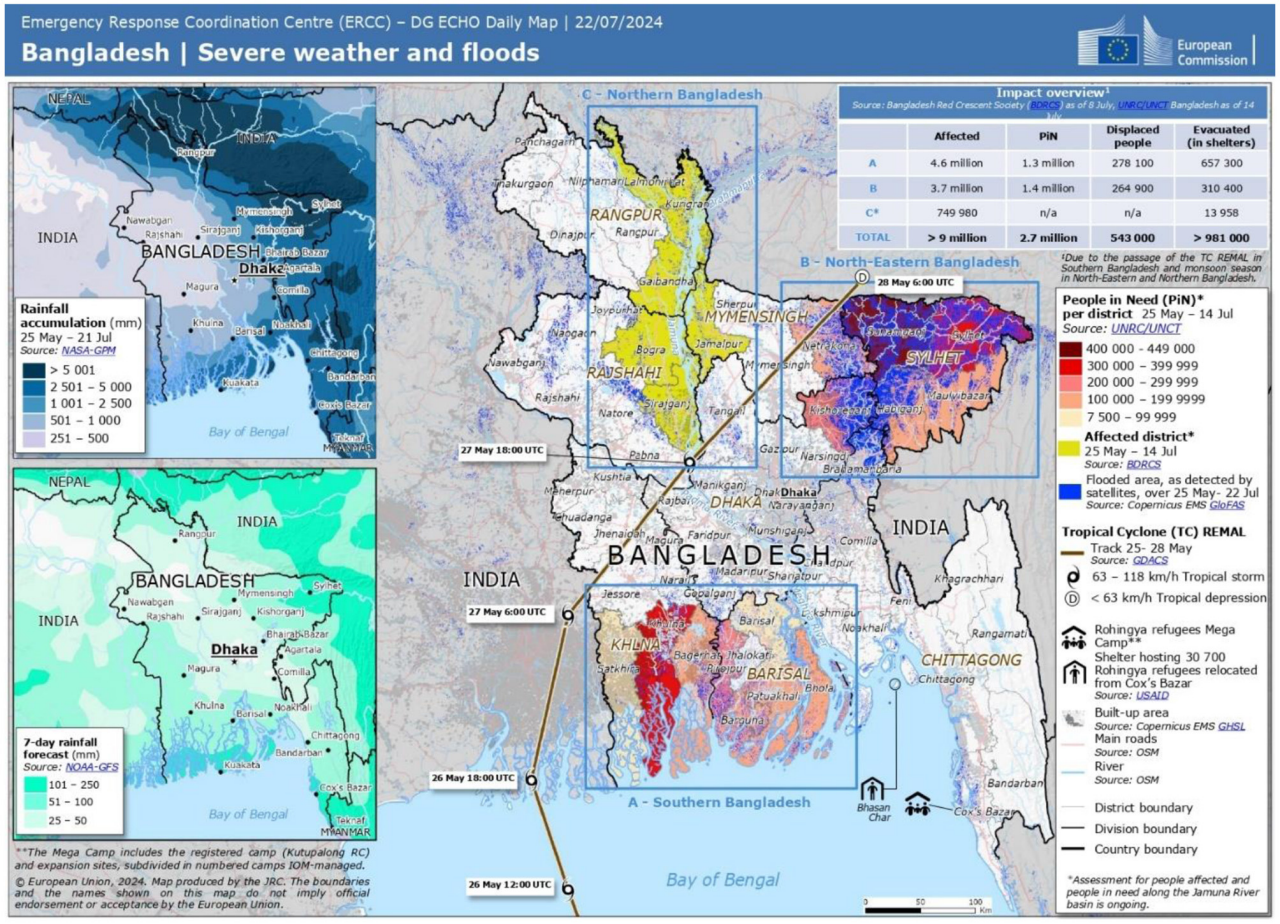
A map released by Global Flood Awareness System (GloFAS News), outlining the flood damage in 2024 Bangladesh (5).

Natural disasters are catastrophic events that occur on a huge scale as a result of meteorological and geological processes that occur naturally on Earth (1). The geographic position of Bangladesh makes it to be among the countries that is most susceptible to the effects of catastrophes at this time (2). Catastrophes such as hurricanes, famine, flooding, earthquakes, severe temperatures, and diseases are among the most common types of natural catastrophes that commonly occur in Bangladesh. The most prevalent catastrophe that occurs in Bangladesh is flooding (3). Bangladesh is under risk of grievous flood during rainy season in every year. A similar situation emerged in 2024 due to heavy rains triggered flooding in 11 states in Bangladesh, affecting ~1 million households (4). Approximately 4.9 million individuals from northeast and southeast regions of Bangladesh are currently marooned, and 20 fatalities have been recorded as a consequence of this devastating flood (4–6). Important services and infrastructure have also taken a major hit as a result of this devastating flood About 26,584 water taps and 62,528 toilets were reported demolished as following this flood. Simultaneously, 2.54 million women have been impacted by flooding across the northeast and southeast regions of Bangladesh (4, 5). Figure 1 shows a map released by Global Flood Awareness System (GloFAS News), outlining the flood damage in 2024 Bangladesh (5).

A study conducted in Bangladesh found that women are considerably more susceptible to catastrophes than men because they are more likely to experience severe impacts as a result of the conditions that make them more sensitive (4). Women experience physical abuse and are frequently displaced from their homes as a result of flooding in every year (1). Challenges in locating suitable housing, nourishment, clean water, and cooking fuel, together with difficulties in upholding menstruation and sanitation, hinder women from fulfilling their customary domestic responsibilities (7). They are also victims of sexual assault and face discrimination when seeking asylum at neighborhood centers (1, 7–9). Women face challenges in the management of their menstruation and keeping proper menstrual hygiene due to insufficient knowledge about menstrual hygiene, cultural and traditional barriers, poor resources and amenities, and limited availability of medical care in catastrophic events (8, 10). Prior to the occurrence of the floods, Bangladesh encountered elevated levels of maternal mortality, particularly in rural regions. The availability of contraceptive and reproductive health care is also affected (2, 11, 12). Moreover, women who are experiencing menstruation encounter difficulties in obtaining sanitary items, which consequently increases their chances of infection (3, 6). Therefore, it is of the utmost importance to focus on menstrual hygiene with the goal to safeguard the health and respect of the flood-affected girls and women in Bangladesh. In order to address the difficulties faced by women and girls during flood, it is crucial to build a healthcare system that is impenetrable to flooding and provide training to health personnel about menstrual control. This will ensure that these amenities can be accessed even during the period of flooding (8, 11, 13). It includes creating a setting where women and girls are esteemed and assisted in keeping their menstrual hygiene with dignity. This involves advocating for gender-responsive strategies in disaster management, which acknowledge and tackle the distinct requirements and susceptibilities of adolescent girls and women during and after floods in Bangladesh (9, 14).

## 2 Methodology

This study employs a case study approach to examine the challenges of menstrual hygiene management (MHM) among women and adolescent girls in flood-affected areas of Bangladesh. It explores socio-cultural barriers, infrastructural limitations, and policy gaps while proposing strategies for integrating MHM into disaster preparedness and response efforts. The research was conducted from June to October 2024 in the most severely flood-affected districts, including Noakhali, Feni, Barisal, Khulna and Satkhira, which are among the most vulnerable regions to climate-induced disasters in Bangladesh.

A qualitative research design was adopted, utilizing a purposive sampling approach (15) to ensure that participants had direct experiences with menstrual hygiene challenges in flood-affected areas. The study included 30 in-depth interviews (IDIs) with affected women and adolescent girls, as well as additional interviews with healthcare workers, NGO representatives, policymakers, and relief workers to capture a multidimensional perspective. Additionally, 12 key informant interviews (KIIs) were conducted with community members, local government officials, and humanitarian actors to discuss socio-cultural stigmas and institutional responses to MHM challenges during floods (9, 16, 17).

Field observations were carried out in temporary shelters and relief centers to assess the availability of menstrual hygiene products and sanitation facilities to enhance external validity (18). Snowball sampling was also employed to reach key informants who could provide critical insights into institutional responses (19). Diversity was ensured in terms of age, socioeconomic status, disability, and marital status, ensuring that the study captured a broad spectrum of experiences. Additionally, case studies were developed to highlight intersectional vulnerabilities, particularly among women with disabilities, older adult women, and widows (20). The triangulation of IDIs, KIIs, field observations, and case studies strengthened the credibility and richness of the findings, providing a holistic understanding of the issue (19, 21).

### 2.1 Data collection process

Data collection was conducted by rigorously trained field researchers using structured interview guides, which were developed in English and translated into Bengali. The interview guides were pre-tested with a small sample of participants from a similar demographic to ensure clarity and cultural appropriateness (15, 18). All interviews and KIIs were audio-recorded with participants' informed consent, and field notes were taken to document non-verbal cues and environmental conditions.

To ensure consistency and reduce interviewer bias, interviewers were trained to ask questions consistently across all interviews (22). They also received cultural sensitivity training, particularly regarding the sensitive nature of menstrual hygiene, to ensure participant comfort and minimize response bias (23). Recognizing the sensitivity of menstrual hygiene management (MHM) in flood-affected areas, all field researchers underwent specialized training on cultural competency, gender-sensitive interviewing techniques, and ethical considerations in qualitative research (24, 25). This training emphasized strategies to build trust with participants, ensure confidentiality, and adopt a non-judgmental approach to discussing menstruation-related challenges. Additionally, interviewers were trained to recognize and mitigate potential discomfort among respondents by employing open-ended, participant-led discussions and ensuring that interviews were conducted in safe, private environments (21, 23).

This training emphasized strategies to build trust with participants, ensure confidentiality, and adopt a non-judgmental approach when discussing menstruation-related challenges. Additionally, interviewers were trained to recognize and mitigate participant discomfort by employing open-ended, participant-led discussions and ensuring that interviews were conducted in safe, private environments (22, 24).

### 2.2 Data analysis process

The audio recordings were transcribed verbatim, translated into English, and uploaded into *Atlas.ti* for qualitative data analysis. An inductive coding approach was employed to identify key themes, with the research team collaboratively developing and refining a coding framework. The coding framework was refined iteratively based on insights gained during the coding process (15, 19).

To enhance rigor, the coding process included inter-coder reliability checks, ensuring that multiple researchers reviewed and agreed on theme development (25). Cohen's Kappa coefficient was calculated to assess inter-coder agreement, ensuring that the findings were consistent and reliable (26). The coding framework was refined through multiple rounds of review. An inductive thematic analysis approach was employed, allowing the research team to revisit and modify codes based on new insights emerging from the data (24, 27, 28). Weekly team meetings were held to discuss evolving themes, resolve inconsistencies, and refine definitions of key concepts, ensuring that the final themes accurately represented participant perspectives and minimized subjective biases. Thematic categories included (a) menstrual hygiene challenges during floods, (b) socio-cultural barriers and stigmas, (c) gaps in infrastructure and policy responses, and (d) coping mechanisms and community-led interventions. Lower-level themes were identified within these broad categories to provide a nuanced analysis of the data. Selected quotes from participants were incorporated to illustrate central themes and highlight divergent viewpoints, ensuring rich and varied insights (29, 30). Lower-level themes were identified within these broad categories to provide a nuanced analysis of the data. Selected participant quotes were incorporated to illustrate central themes and highlight divergent viewpoints, ensuring rich and varied insights (20, 29, 31).

### 2.3 Ethical considerations

This study was reviewed and approved by the Institutional Review Board (IRB) of the Department of Sociology, Gopalganj Science and Technology University (GSTU), Gopalganj-8100, Bangladesh. The approval number for this research is GSTU/SOC/IRB/2025/038. All procedures performed in this study involving human participants were in accordance with the ethical standards of the IRB at GSTU and with the 1964 Helsinki Declaration (32) and its later amendments or comparable ethical standards.

#### 2.3.1 Informed consent

Written informed consent was obtained from all participants prior to their inclusion in the study. For vulnerable groups, such as women with disabilities, older adult women, and widows, the consent process was conducted orally and in writing, with additional explanations provided in local dialects to ensure full comprehension. Trained female researchers facilitated discussions in private and secure settings to respect participants' comfort and autonomy.

#### 2.3.2 Protection of sensitive data

Confidentiality was rigorously maintained by assigning pseudonyms to all respondents. All identifying information was anonymized during transcription and analysis. Data were stored securely on password-protected devices, with access restricted to authorized research personnel only.

#### 2.3.3 Special ethical safeguards for vulnerable groups

Recognizing the heightened risks for women with disabilities, older adult women, and widows, additional ethical safeguards were implemented. Community facilitators were engaged to provide culturally sensitive support, ensuring that participation was voluntary and free from coercion. Psychosocial support referrals were arranged for participants who expressed distress during discussions.

To enhance cultural sensitivity, local facilitators assisted with translation and community engagement throughout the research process. Findings were triangulated with secondary literature, policy reports, and international case studies to enhance validity and contribute to the broader discourse on disaster management, public health, and gender equity.

## 3 Results

### 3.1 Theme 1: barriers to menstrual hygiene management during floods

Floods disrupt not only the physical environment but also the basic human needs for health and hygiene. In flood-affected areas, the barriers to menstrual hygiene management (MHM) become particularly pronounced. One of the most immediate barriers is the scarcity of sanitary products. In emergencies, resources are limited, and sanitary products are often deprioritized in favor of food, water, and shelter. This scarcity leaves women with little choice but to resort to unhygienic materials such as rags, leaves, or old cloth.

“During the floods, it was impossible to find any sanitary pads. I had to use cloth, but it wasn't enough. We were stuck in shelters with no access to basic hygiene materials.” (*Interviewee 1; 25 years, Noakhali*)

The difficulty of acquiring sanitary materials is compounded by the disruption of supply chains. Flooding often damages roads, transportation systems, and retail outlets, further impeding the availability of sanitary products. Even in cases where aid is provided, it is frequently short-term and insufficient, failing to meet the long-term menstrual hygiene needs of women and girls in these affected communities.

“We had to rely on whatever we could get from relief supplies, but it wasn't enough for our needs. Sometimes, we had to use leaves and newspapers.” (*Interviewee 21; 30 years, Satkhira*)

In addition, women in flood shelters or temporary camps are faced with the challenge of limited privacy. Cultural norms surrounding menstruation in many flood-affected areas, especially rural settings, restrict women's movement and access to private spaces. The absence of designated spaces for changing menstrual products leads to discomfort and often results in unsanitary practices. In these environments, where access to clean water is also compromised, women may be unable to properly clean their menstrual products or their bodies, leading to a significant increase in health risks, including infections.

### 3.2 Theme 2: cultural and social barriers to MHM

Cultural and social stigma surrounding menstruation is one of the most significant barriers women face in managing their menstrual hygiene, particularly during natural disasters such as floods. In many communities, menstruation is seen as a taboo subject, and women are taught to feel shame or embarrassment about their menstrual cycles. This social stigma often prevents women from discussing their menstrual health openly, resulting in a lack of access to accurate information or resources on proper menstrual hygiene management.

“I was embarrassed to ask for sanitary pads. It feels like we're not supposed to talk about it, so I just used whatever I could find.” (*Interviewee 15; 22 years, Barisal*)

During emergencies like floods, this cultural barrier is exacerbated. Women may be reluctant to ask for menstrual hygiene products, discuss their needs, or report challenges due to the fear of being stigmatized. In flood shelters or displaced communities, where space is often shared by multiple families, privacy becomes a luxury. The absence of private spaces to change or dispose of menstrual products forces many women to manage their menstruation in conditions that are both unsanitary and emotionally distressing.

“In our village, menstruating women are not supposed to be seen outside. During the floods, there was nowhere private to change, and I felt very ashamed.” (*Interviewee 6; 28 years, Khulna*)

Social expectations that menstruating women should isolate themselves, stay away from public places, or avoid certain activities are magnified during a crisis. These gendered expectations lead to further isolation and a lack of community support for managing menstrual hygiene. The compounded impact of cultural stigma and restricted access to resources places significant psychological and physical strain on women, making it more difficult to maintain dignity and health during the flood crisis.

### 3.3 Theme 3: infrastructural and environmental challenges

The environmental and infrastructural challenges posed by floods create a perfect storm for poor menstrual hygiene practices. Flooding compromises the infrastructure of affected areas, including roads, sanitation facilities, and the water supply. The breakdown of essential infrastructure leads to the contamination of water sources, rendering access to clean water virtually impossible. With access to clean water already a challenge, using menstrual hygiene products becomes an even greater risk, as the materials used to manage menstruation may get contaminated.

“I was embarrassed to ask for sanitary pads. It feels like we're not supposed to talk about it, so I just used whatever I could find.” (*Interviewee 5; 22 years, Feni*)

Sanitation facilities, which are already often inadequate in flood-prone areas, are further compromised by floodwaters. Toilets, bathrooms, and disposal systems become submerged, leaving women with few choices but to resort to open spaces or unsanitary environments. In many cases, women are forced to manage menstruation in flooded conditions, leading to increased exposure to waterborne diseases, infections, and other health risks.

“In our village, menstruating women are not supposed to be seen outside. During the floods, there was nowhere private to change, and I felt very ashamed.” (*Interviewee 11; 28 years, Noakhali*)

Beyond the immediate health concerns, the environmental impact of poor menstrual hygiene management during floods is significant. The disposal of menstrual products in flood-affected areas, particularly those made from non-biodegradable materials like plastic, contributes to environmental degradation. The failure to address menstrual hygiene management in the context of disaster preparedness and response exacerbates the long-term environmental damage in these already vulnerable communities.

### 3.4 Theme 4: gaps in menstrual hygiene education and awareness

The lack of education and awareness regarding menstrual hygiene management (MHM) in flood-affected areas is a major challenge. In many rural and impoverished regions, particularly in Bangladesh, menstruation remains a taboo subject, and comprehensive education on menstrual hygiene is lacking. Without proper education, women and girls may be unaware of the risks associated with poor menstrual hygiene practices or the methods to manage menstruation effectively, especially during floods.

“I didn't know that reusing cloth could cause infections, but we didn't have any other choice.” (*Interviewee 2; 18 years, Satkhira)*

When floods occur, the situation worsens due to the suddenness and unpredictability of disasters. People are already overwhelmed by the loss of homes, access to food, and the upheaval of their lives. In these conditions, basic needs take precedence, and menstrual hygiene often becomes a secondary concern. This lack of education and preparedness leads to unhealthy coping mechanisms, such as the use of unhygienic materials, which increase the risk of infections, rashes, and long-term health complications.

“Menstruation is never discussed in our community. During the floods, we had no guidance on how to manage it safely.” (*Interviewee 10; 36 years, Feni*)

Moreover, misinformation and cultural myths about menstruation further perpetuate poor practices. For instance, in some communities, menstruating women are believed to be “unclean” and may be restricted from entering certain areas, affecting their ability to access clean water and private spaces to change. A lack of information leads to the reinforcement of harmful stereotypes and practices, creating a cycle of neglect and poor health.

### 3.5 Theme 5: coping strategies for menstrual hygiene during floods

In the absence of ideal resources, women in flood-affected areas develop coping strategies to manage menstruation under adverse conditions. While these strategies reflect resilience, they often come with significant health and psychological risks. One common coping mechanism is the reuse of cloth, which is often not properly sanitized. Cloths are used repeatedly, increasing the risk of infections such as urinary tract infections (UTIs), vaginal infections, and rashes.

“We had no choice but to use old clothes. They weren't clean, but we couldn't go without anything.” (*Interviewee 11; 23 years, Barisal*)

Women also rely on alternative materials such as leaves, rags, or even newspapers. While these materials may be available in the short term, they are not designed for menstrual hygiene and do not provide the necessary absorbency or protection against leaks. The lack of adequate options forces women to make do with what is available, often compromising their health in the process.

“I had to use newspaper because we didn't get any pads or cloth. It was uncomfortable, but we just had to make do.” (*Interviewee 22; 33 years, Feni*)

In addition to the physical challenges, these coping strategies come with emotional and psychological burdens. The lack of privacy, combined with the social stigma surrounding menstruation, results in feelings of embarrassment, shame, and frustration. Women may avoid seeking help or discussing their needs with others, leading to increased isolation and mental distress during an already traumatic time.

### 3.6 Theme 6: impact of menstrual hygiene on health and wellbeing

The physical and emotional health impacts of inadequate menstrual hygiene management during floods are profound. On the physical side, poor menstrual hygiene can lead to a range of health complications, including infections, rashes, and more serious conditions such as reproductive tract infections (RTIs). The use of contaminated materials or unhygienic products like cloth, leaves, and rags increases the likelihood of such infections. When women and girls are unable to clean themselves properly, they are more vulnerable to conditions like yeast infections, bacterial infections, and urinary tract infections (UTIs).

“I felt so embarrassed. I couldn't clean myself properly, and I was worried about getting sick.” (*Interviewee 13; 27 years, Noakhali*)

Psychologically, the inability to manage menstruation in a dignified and hygienic manner can have a severe emotional impact. Feelings of shame, embarrassment, and distress are common, especially when women are unable to access private spaces for changing their menstrual products. This can lead to stress, anxiety, and a sense of powerlessness, which exacerbates the trauma of living through a natural disaster.

“My health deteriorated because I couldn't manage menstruation properly during the flood. I started having infections.” (*Interviewee 19; 38 years, Khulna*)

Furthermore, the long-term health consequences of poor menstrual hygiene can affect women's overall wellbeing, leading to chronic health issues that require medical intervention. The impact on women's mental health is equally significant, as the experience of menstruating in public, in unsanitary conditions, and with inadequate resources can lead to a sense of isolation and helplessness.

## 4 Discussion

In Bangladesh, people often don't receive an adequate amount of relief during floods due to limited resources. After periods of calamity, unsanitary and severely unhealthy conditions emerge in the flood-affected areas (1). Especially, women are disproportionately impacted by floods, experiencing significant deprivation during times of calamity. Insufficiently clean water and limited availability of hygiene and sanitation facilities during catastrophes pose health risks specifically for women (33). Menstrual hygiene management is overlooked in overall health objectives. They lack medical amenities pertaining to menstruation hygiene. Ensuring gynecological health requires the regular replacement of cloths/rags or pads every 4 h to preserve menstrual hygiene (2). But acquiring sanitary pads is frequently not affordable in flood-affected areas in Bangladesh (8, 16). Therefore, women reluctantly utilize discarded clothes as absorbent materials for blood. Due to inadequacy of knowledge and resources, they use floodwater for laundering their clothes to clean the blood. Unfortunately, it is considered disgraceful for them to launder or air-dry the clothes in the presence of male individuals in shelter houses. Our findings also align to previous research, girls in flood-affected regions face challenges in managing menstrual hygiene due to the scarcity of water, limited privacy, and inadequate drying facilities (8, 18, 23).

**Figure 2:**
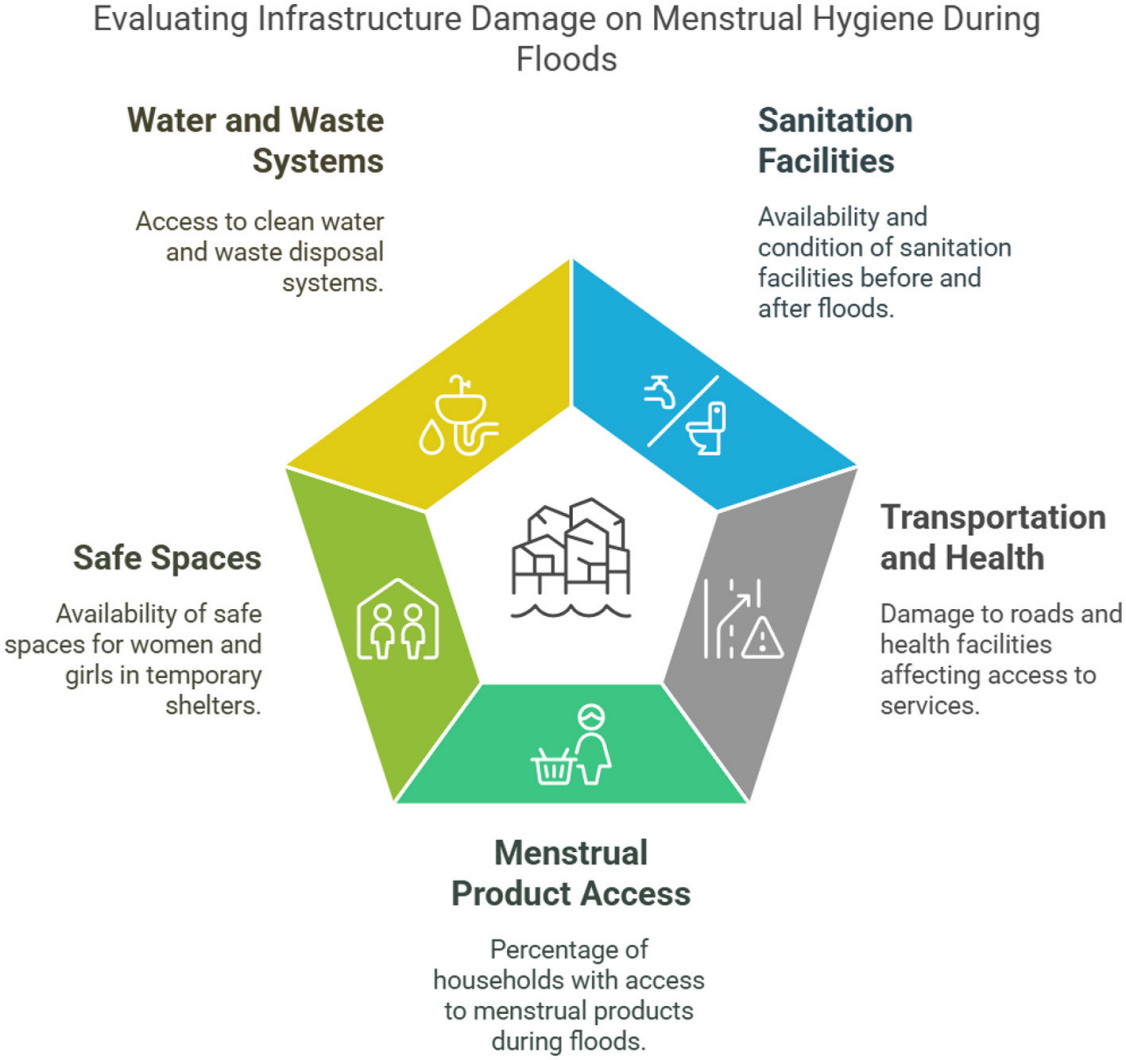
Evaluating infrastructure damage on menstrual hygiene during floods (Authors illustration).

In Bangladesh, regulating menstrual hygiene is a significant difficulty for many women and girls during non-flooding times. Because sanitary pads are still expensive and difficult to get in rural areas, many women are forced to use reusable, frequently improperly sanitized cloths (19, 32). Lack of sufficient restroom facilities at home often compromises privacy for managing menstruation, especially in homes with low incomes (20, 34, 35). The problems are further compounded by the lack of knowledge and instruction regarding menstrual health, which leaves women susceptible to infections and other health hazards even in everyday situations (22, 23). This gap is worsened by limited menstrual health education, which perpetuates harmful practices and increases the risk of infections and other health issues (19, 24). Even during non-crisis periods, many women are compelled to use inadequate alternatives like old cloths for menstrual management, often reusing them without proper cleaning due to limited access to safe and affordable menstrual products (25, 36). This issue is particularly acute in rural regions, where infrastructure for private sanitation facilities at homes or schools is scarce (20, 37). The lack of clean water and effective disposal systems for menstrual waste not only heightens environmental concerns but also poses significant health risks (5, 25, 38). In many cases, the high cost and limited availability of sanitary pads force women to rely on reusable cloths that are frequently unsafely sanitized (26, 39). Furthermore, inadequate restroom facilities at home often compromise privacy for menstruation management, especially for women from low-income households (29, 34, 35). Compounding the issue is the widespread lack of awareness and education on menstrual health, leaving women vulnerable to infections and other health complications in their daily lives (27, 32, 33). These underlying obstacles are exacerbated by floods, resulting in a worsened health crisis for women (see Figure 2).

Inadequacy about menstrual hygiene among people in Bangladesh also makes notable resistance to maintaining menstrual hygiene (36, 40). Most of the women who took shelter in centers due to floods didn't know anything about frequent pad replacement or methods of disposal for old pads (16, 25, 35). This limited knowledge about menstrual hygiene management among women makes it difficult for the service providers to propagate awareness about menstrual hygiene. Moreover, social and cultural stigmas regarding menstrual hygiene also become prominent because of a lack of knowledge (25, 26, 29). Openly discussing menstruation was prohibited in the patriarchal society. Girls also experienced reticence while discussing this matter with their parents. Insufficient understanding of menstruation hygiene renders individuals more susceptible (18, 38). There was a lack of concern and attention toward women's menstrual cycles and a disregard for solving their issues. Hence, adolescent girls in Bangladesh faced conservative social stigma regarding menstruation, which was especially prominent in flood-affected areas (15, 16, 18).

In the South Asian context, a grievous cultural taboo was also found about maintaining menstrual hygiene. It was elucidated that when a girl menstruates for the first time, she is denied any food until the fourth day and prohibited from interacting with male members (40). Adolescent girls and women are required to consistently adhere to certain menstrual taboos on a monthly basis during their menstrual cycle. These taboos not only limit their mobility but also restrict their ability to maintain hygiene, such as properly washing and drying reusable cloths (16, 41). This ritual also plays a significant role while managing menstrual hygiene management during disasters like floods (12).

**Figure 3:**
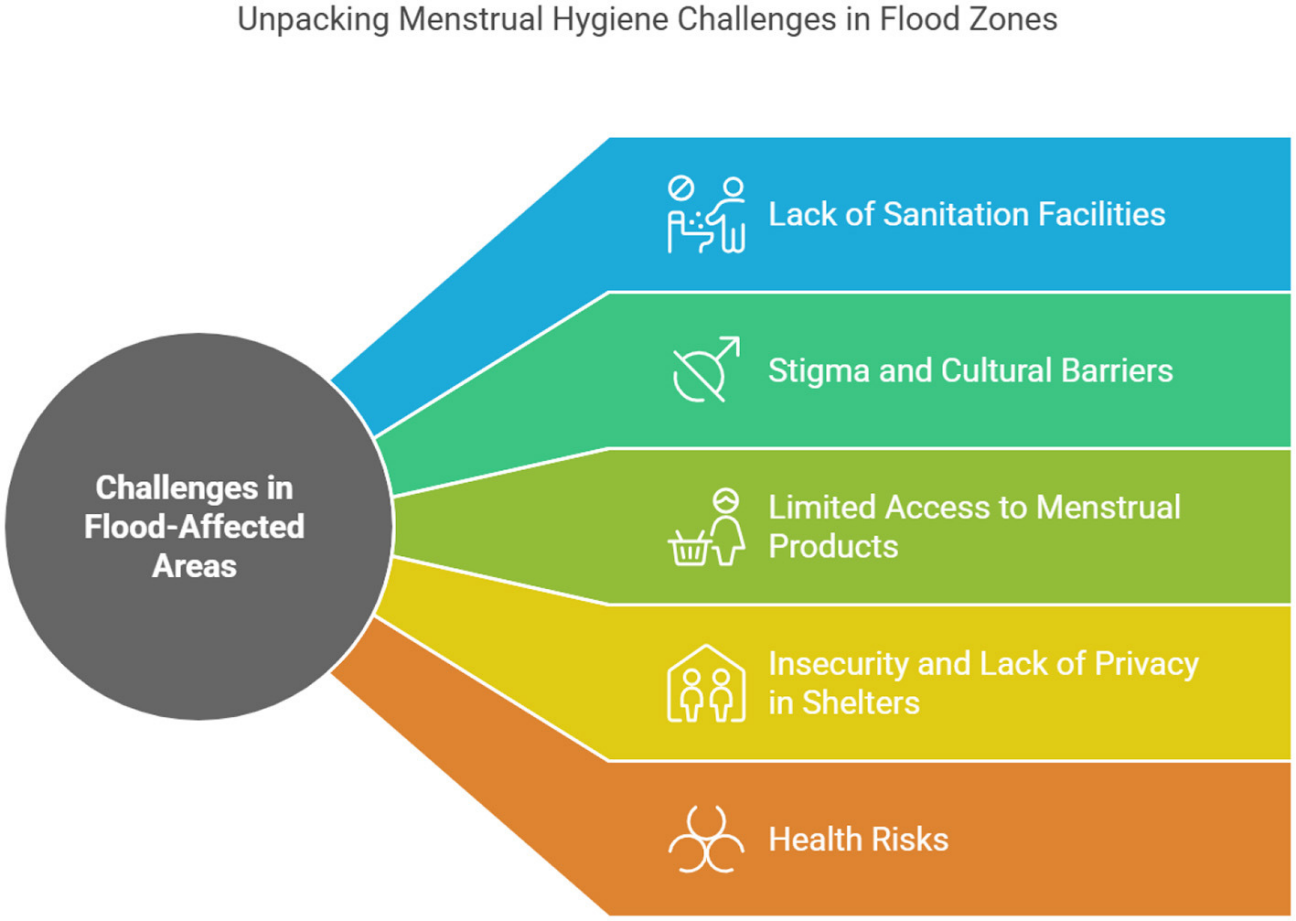
Menstrual hygiene challenges in flood-affected zones in Bangladesh (Authors illustration).

Menstrual hygiene management (MHM) necessitates convenient access to secure and secluded sanitation and pure water services. In low-income countries (LMICs), it was frequently impaired in the challenging settings of an asylum during floods. Due to the inaccessibility of residences and reservoirs during floods, women were unable to take a bath for extended periods (37). Some individuals also stated that they never got soap to freshen themselves. During floods, toilets, and other cleanliness and sanitation amenities are severely affected when they become drowned in low-lying locations (8). Following this situation, they had to seek refuge and utilize toilets in the relief shelters (12). Moreover, gender-segregated toilets weren't available in the relief centers. The combination of these variables is a formidable obstacle to upholding menstrual hygiene amidst the floodwaters (34). Women in flood-affected communities may face challenges in communicating with predominantly male rescue workers due to the influence of patriarchal society. In addition, distribution sites pose a safety risk for women due to the presence of huge crowds and the potential for sexual assault (22, 23). In low middle income countries, the crisis shelters lacked a focus on women's needs, resulting in a disregard for their protection, privacy, and health concerns. Girls refrained from using washroom amenities while dislocation because of an abundance of males (3). Toilets in displacement camps were overcrowded, unsanitary, and did not have adequate trash disposal facilities. In addition, the substantial gaps in washrooms between the bamboo walls that allow for visibility, and the lack of a door lock that compromises security (36) (see Figure 3).

Moreover, the lack of secure and secluded spaces for changing menstruation products was a big issue. This was particularly problematic because the community washrooms were usually located outside the tents, which meant that menstruating women had to transport their menstrual products through the campsite (15, 19). Furthermore, numerous toilets were devoid of a hinge, which resulted in women feeling unsafe while exchanging their menstrual products (20, 36, 41). The absence of privacy in evacuation centers was a significant worry. Even, they expressed a feeling of ashamed about MHM kits being supplied publicly alongside other non-food goods. Women and girls felt about the loss of my dignity while confronting those impediments in relief centers (37).

## 5 Policy recommendations

Few policies and ideas were also taken by Bangladesh government to vindicate menstrual hygiene management system (39). In order to develop an approach to manage menstrual hygiene in catastrophes, the first step is to recognize and deal with prejudiced ideas around this issue, and then deploy feasible options that can substitute them (35). Programs and trainings are organized to educate women and teenage girls about menstruation health and cleanliness, with the aim of raising awareness. This subject needs to be incorporated into the educational curriculum and mandated for both male and female students for the purpose to foster awareness and comprehension of the issue (8, 23). The mainstream media as well as social platforms have the ability to significantly contribute to increasing awareness within the general population about menstrual hygiene. It is crucial in today's world to utilize technologies aiming to promote consciousness and campaigns (38). It is also important to enable males to discuss the subject of menstruation without embarrassment as the majority of health care providers are men. Specialized orientation courses for frontline staffs in humanitarian response can accomplish this (18, 25).

Government or organizations should enhance the supply of essential products like sanitary pads, fresh linens etc. for maintaining menstrual hygiene in flood affected areas (8, 25). The Menstrual Health management packages need to be distributed on time (19, 38). Leaflets should be accompanied by Menstrual Hygiene Management (MHM) kits that provide comprehensive guidance on menstrual cycle monitoring, adequate hygiene practices, and the usage and disposal of sanitary pads (40). The development of novel sanitary products, such as affordable, eco-friendly pads and menstrual cups, has been promoted by multiple efforts (35). Along with that, a regional market system can be established so that women could get vital goods at inexpensive prices (7). Establishing a medical clinic in flood affected areas just for women and offering female gynecologist and nurses can also help to meet the demands considering the menstrual hygiene management (8). Communal washrooms must be designed to accommodate menstrual hygiene management (MHM) activities such as washing, changing, and disposing of menstrual products (24, 42).

**Figure 4:**
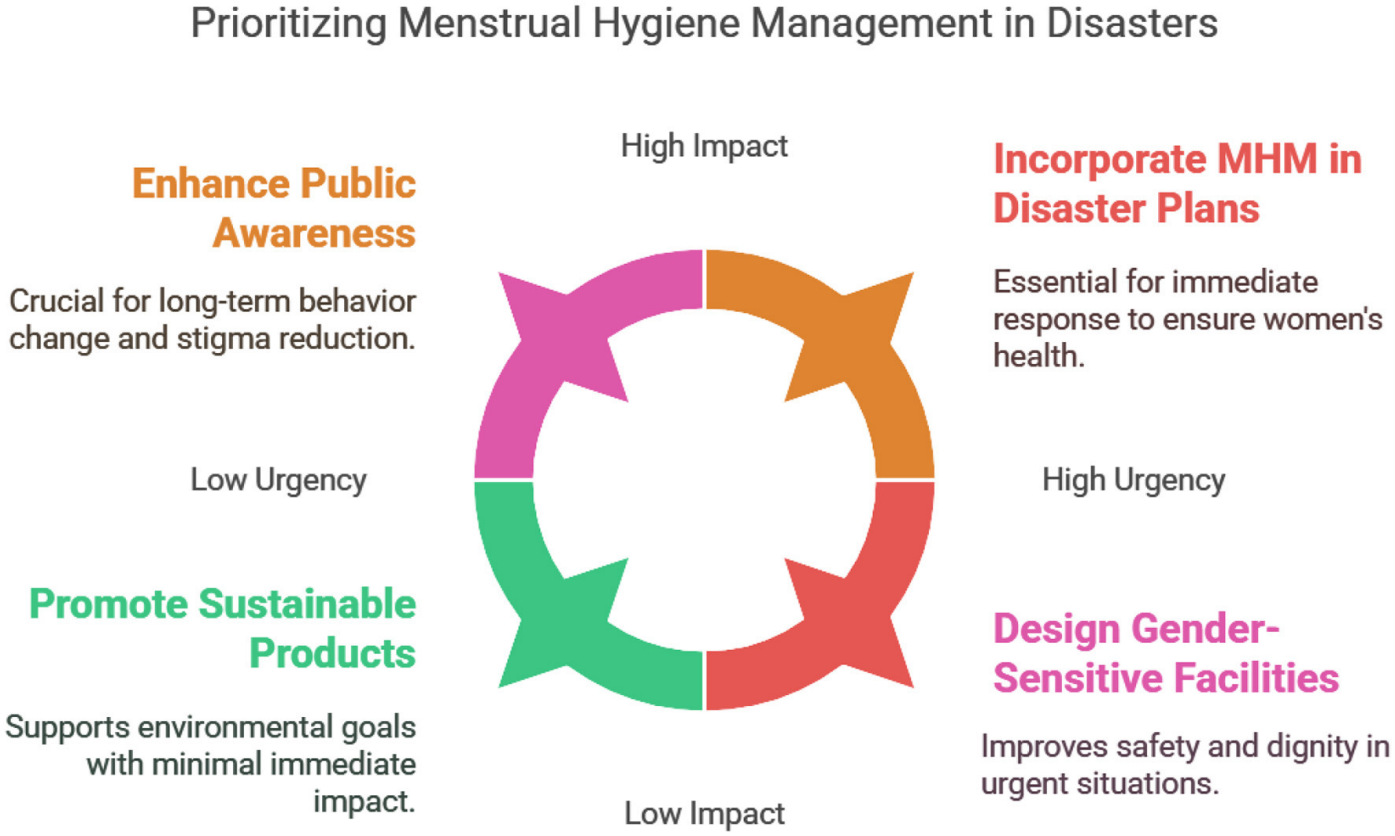
Infographics illustrating proposed solutions in prioritizing MHM in disasters (Authors illustration).

A noteworthy example comes from the 2010 floods in Pakistan, where the government, in collaboration with international humanitarian organizations, implemented measures to ensure access to menstrual hygiene products and education. Mobile health units were deployed to provide sanitary supplies and raise awareness on menstrual health, especially in remote and marginalized communities (39). In India, particularly in flood-prone states like Uttar Pradesh and Bihar, the government and non-governmental organizations (NGOs) have worked together to provide MHM support during natural disasters. Efforts such as the distribution of sanitary kits and the inclusion of menstrual hygiene in disaster management plans have proven effective in addressing the unique needs of women and girls during flood events (18, 20, 42). This approach to disaster response can offer valuable insights for Bangladesh in terms of expanding mobile health services and integrating MHM into disaster preparedness. To maintain menstrual hygiene management during natural disasters, it is necessary to get the least amount of water that is needed for their personal needs of women and girls. Women's burden regarding this, will be dwindled when they have accessibility to clean water resources and services that offer a suitable, secure, and consistent flow of water in order to cope with sanitation concerns (see Figure 4).

### 5.1 Enhance public awareness and education on MHM during emergencies

Increase public awareness about menstrual hygiene management during floods and other natural disasters through media campaigns, community programs, and school-based education. Many women and girls lack proper knowledge of how to manage menstruation during crises, leading to health risks and social stigma. Educational campaigns should include information on how to use and dispose of sanitary products safely and hygienically during floods. Partner with local NGOs, the Ministry of Health, and educational institutions to run awareness programs at community centers, schools, and shelters.

### 5.2 Incorporate MHM as a standard component in disaster preparedness plans

Include menstrual hygiene products (e.g., sanitary pads) as a mandatory component in emergency relief kits distributed during floods. The lack of sanitary products in flood-affected areas is one of the primary barriers to MHM during disasters. Including menstrual hygiene products in relief packages would ensure that women and girls have access to necessary supplies, reducing health risks and social discomfort. Collaborate with disaster management agencies, the Ministry of Disaster Management and Relief, and humanitarian organizations to ensure that MHM supplies are included in all disaster relief materials.

### 5.3 Design and implement gender-sensitive sanitation facilities in shelters

Develop and install safe, private, and hygienic sanitation facilities in temporary shelters during flood events, specifically tailored to meet the needs of women and girls. The absence of private and clean spaces for menstruation management in flood shelters leads to social stigma and mental health issues. Providing women-friendly latrines and washing areas can significantly improve dignity and safety for women and girls in shelters. Disaster response teams should collaborate with WASH (Water, Sanitation, and Hygiene) experts to ensure that flood shelters include gender-sensitive facilities, such as private latrines, waste disposal for menstrual products, and sufficient lighting.

### 5.4 Promote research on local MHM practices and needs

Conduct further research on the specific menstrual hygiene practices, cultural norms, and challenges faced by women and girls during floods in Bangladesh, focusing on different regions and communities. Research on the local context, including cultural attitudes toward menstruation, types of sanitary products used, and existing coping mechanisms, can inform more effective interventions. Understanding the socio-cultural dynamics will help tailor responses to specific community needs.

Universities, research institutions, and NGOs should prioritize ethnographic studies, surveys, and participatory research methods to gather data from affected communities on their MHM needs and practices during disasters.

### 5.5 Integrate MHM into national disaster management policies

Include menstrual hygiene management in the national disaster management policies and frameworks as a critical element of women's health and wellbeing during emergencies. Despite the recognition of gender issues in disaster response, MHM is often overlooked in national disaster management frameworks. Formalizing MHM as part of the official disaster management plan would ensure its inclusion in the distribution of resources and services during floods. Advocate for the revision of the National Disaster Management Policy to include specific provisions for MHM, such as the distribution of sanitary products, establishment of safe spaces, and provision of relevant health services.

### 5.6 Improve post-flood MHM support services

Establish long-term support services for women's menstrual hygiene needs following floods, including access to health care, sanitation services, and menstrual products. After the initial relief phase, women may still face challenges in managing menstrual hygiene, especially in rural and remote areas. Post-flood support services should include continued access to health services and sanitary products, as well as psychosocial support to help women cope with the trauma of the disaster. Coordinate with local health centers, NGOs, and community organizations to provide continued menstrual hygiene support after the floodwaters recede, ensuring that women and girls can access sanitary products and health services.

### 5.7 Promote sustainable and eco-friendly menstrual hygiene products

Advocate for the use of eco-friendly and sustainable menstrual hygiene products in flood relief efforts. The environmental impact of disposable sanitary products, especially in flood-affected areas, can be significant. Promoting the use of reusable or eco-friendly menstrual products (such as menstrual cups or cloth pads) can reduce waste and provide a more sustainable solution during disasters. Partner with NGOs and manufacturers of eco-friendly menstrual hygiene products to distribute these items in flood relief packages and educate communities on their benefits.

### 5.8 Implement community-based education and awareness programs

In Bangladesh, community-based education and awareness programs have been successfully implemented in various public health initiatives, such as maternal health campaigns and immunization drives. For instance, the Government of Bangladesh, in collaboration with NGOs like BRAC and WaterAid, has launched menstrual health awareness programs in rural and flood-prone areas. These programs emphasize community engagement, using trained facilitators to conduct door-to-door awareness sessions, ensuring that marginalized women and girls receive critical information on menstrual hygiene.

In addition to these efforts, it is crucial to involve male community members and religious leaders in dismantling menstrual taboos. Engaging men and boys in awareness campaigns is essential for breaking the cycle of silence and stigma surrounding menstruation. For example, integrating discussions on menstrual health within community meetings, where men are present, can encourage them to share responsibility for addressing menstrual hygiene. Male community champions can play a pivotal role in challenging negative attitudes toward menstruation by serving as advocates and role models for other men and boys.

Furthermore, religious leaders have a significant influence in many Bangladeshi communities. By involving them in educational campaigns, we can tap into their capacity to spread positive messages about menstruation, normalizing the topic in a culturally sensitive manner. Training religious leaders on menstrual hygiene can equip them to address misconceptions and promote open dialogue in mosques and community gatherings. They can play a crucial role in reframing menstruation as a natural process rather than something to be stigmatized.

Initiatives like UNICEF's Menstrual Hygiene Management (MHM) program in Bangladesh have also integrated storytelling and community dialogues to address stigma, encouraging open discussions among adolescents, caregivers, and families. These programs emphasize shared responsibility between genders, with both men and women participating in discussions that challenge cultural taboos. Distribution of culturally appropriate educational materials, such as illustrated booklets and videos in Bengali, has been effective in shifting perceptions and promoting menstrual health education. These real-life policy approaches highlight the effectiveness of grassroots awareness campaigns in tackling menstrual stigma and improving hygiene practices in disaster-affected areas.

### 5.9 Foster public-private partnerships for MHM solutions

Strengthen collaborations between government agencies, the private sector, and non-governmental organizations (NGOs) to improve the availability and accessibility of menstrual hygiene products during floods. The private sector, particularly manufacturers of sanitary products, can play a vital role in ensuring a steady supply of menstrual hygiene products during floods. Public-private partnerships can help expand distribution networks and create innovative solutions to meet MHM needs. Establish partnerships between the government, local businesses, and NGOs to stockpile and distribute menstrual hygiene products in flood-prone areas. Develop collaborative strategies for sustainable access to sanitary products during disasters.

One of the most important aspects of providing vital reproductive health treatments during times of crisis is the Minimum Initial Service Package, also known as the MISP. Through its implementation, sexual assault can be prevented, needs of adolescents can be met (27). A number of different actions that might be taken to promote the empowerment of local women and lessen their susceptibility to any potential calamities in the future. Among these solutions is the implementation of legislation that have legal backing and have the potential to prevent assaults on women (7).

## 6 Study limitations and future research directions

This study is limited by its focus solely on floods, which restricts a comprehensive understanding of menstrual health management in the context of other disasters or stable periods. While the findings offer valuable insights into the challenges faced by women and adolescent girls during flood events, they may not fully capture the complexities of menstrual health management in other emergency situations, such as cyclones, droughts, or prolonged displacement. Additionally, the reliance on qualitative data, while offering depth and rich narratives, limits the generalizability of findings across broader populations.

Future research should aim to explore menstrual health management in diverse disaster contexts, including long-term displacement and humanitarian crises, to develop more inclusive and adaptable interventions. Incorporating mixed-method approaches by integrating qualitative narratives with secondary quantitative data could provide a more comprehensive understanding of the scope and scale of menstrual health challenges. Comparative studies across different disaster-prone regions may also help identify common patterns and region-specific barriers, contributing to the development of targeted policy recommendations.

## 7 Conclusion

Bangladesh is recognized as one of the most disaster-prone countries globally, frequently impacted by natural disasters, with floods being one of the most common and recurrent challenges. This study specifically focused on the challenges that women and girls face in maintaining menstrual hygiene during flood events, when access to essential resources, safe environments, and sanitation facilities is severely compromised. However, it is important to acknowledge that similar challenges also arise in other natural disasters, such as cyclones, droughts, and other climate-induced events, which were outside the scope of this study. Additionally, issues that persist during non-crisis periods, such as the high cost of menstrual products, inadequate sanitation facilities, and limited access to hygiene resources, continue to exacerbate menstrual hygiene challenges and need to be addressed as part of long-term policy interventions.

To effectively address the specific challenges observed during floods, a comprehensive and multi-faceted approach is necessary. This approach should focus on raising awareness, ensuring the affordability and accessibility of menstrual hygiene products, establishing secure and private sanitation facilities, and embedding menstrual health care into disaster preparedness and response frameworks. Importantly, broadening the scope to include other natural disasters and non-crisis periods is essential for developing more inclusive, adaptable strategies that prioritize the dignity, health, and wellbeing of women and girls across various contexts.

Recognizing menstrual hygiene management (MHM) as a fundamental human right is pivotal to safeguarding the dignity, safety, and health of women and girls during crises and beyond. By integrating MHM into disaster management frameworks, policy interventions can contribute to enhancing gender equity, reducing stigma, and ensuring that women and girls are equipped to maintain their health and dignity in the face of any natural disaster. Last but not the least, shift toward more inclusive and sustained efforts that address both emergency and non-emergency challenges will create a stronger foundation for achieving lasting improvements in menstrual hygiene management and overall gender equity in Bangladesh.

## Data Availability

The original contributions presented in the study are included in the article/supplementary material, further inquiries can be directed to the corresponding author.
